# Lung flute improves symptoms and health status in COPD with chronic bronchitis: A 26 week randomized controlled trial

**DOI:** 10.1186/s40169-014-0029-y

**Published:** 2014-09-23

**Authors:** Sanjay Sethi, Jingjing Yin, Pamela K Anderson

**Affiliations:** 1 VA WNY Health Care System, 3495 Bailey Avenue, Buffalo 14215, NY, USA; 2 University at Buffalo, Suny, Buffalo, NY, USA

**Keywords:** Mucus clearance, COPD, Oscillatory device, Chronic bronchitis

## Abstract

**Background:**

Chronic obstructive pulmonary disease (COPD) is characterized by mucus hypersecretion that contributes to disease related morbidity and is associated with increased mortality. The Lung Flute® is a new respiratory device that produces a low frequency acoustic wave with moderately vigorous exhalation to increase mucus clearance. We hypothesized that the Lung Flute, used on a twice daily basis will provide clinical benefit to patients with COPD with chronic bronchitis.

**Methods:**

We performed a 26 week randomized, non-intervention controlled, single center, open label trial in 69 patients with COPD and Chronic Bronchitis. The primary endpoint was change in respiratory symptoms measured with the Chronic COPD Questionnaire (CCQ). Secondary endpoints included health status, assessed by the St. George Respiratory questionnaire (SGRQ), BODE (Body-Mass Index, Airflow Obstruction, Dyspnea, and Exercise Capacity)

index score and exacerbation frequency.

**Results:**

While the control patients did not demonstrate any significant changes in the primary endpoint (CCQ change at 26 weeks of +0.01, p = 0.8), a trend (p = 0.08) to decrease (improvement) in the CCQ (-0.23 at 26 weeks) was seen with the Lung Flute. Furthermore, a significant improvement in the symptom domain of the CCQ was seen only with the lung flute (-0.42, p = 0.004). Health status (SGRQ) improvement, was also only seen with the Lung Flute (-3.23, p = 0.03). The BODE score increased in the control group (3.31 at baseline, 4.14 at 26 weeks), however it remained stable in the Lung Flute arm (3.16 at baseline and 26 weeks), with the changes from baseline being significantly different between the 2 arms (p = 0.01). There was a trend for less exacerbations in the Lung Flute group (p = 0.07). Adverse effects were minor, with only 1 patient discontinuing treatment because of lack of efficacy. Serious adverse effects seen were all determined to be unrelated to the device use.

**Conclusions:**

The Lung Flute is a safe and effective treatment in COPD with chronic bronchitis, providing a wide array of benefits.

**ClinicalTrials.gov Identifier:**

NCT01186822

## Background

Mucus hypersecretion and impaired mucociliary clearance is prevalent in many patients with COPD, and contributes significantly to the morbidity and mortality of this disease [1],[2]. In spite of the need for efficacious, convenient and safe treatment for mucus hypersecretion, current choices are few with limited data to support their efficacy in COPD [3],[4]. The Lung Flute is a new small self-powered audio device that has been classified by the Food and Drug Administration (FDA) to the family of Oscillatory Positive Expiratory Pressure (OPEP) devices, which includes the Flutter® and the Acapella® [5],[6]. However, unlike traditional OPEP devices that use oscillatory back pressure, the Lung Flute has a unique mechanism of action based on acoustic energy. When blown in to with an exhalation vigorous enough to make the reed oscillate, the Lung Flute generates a sound wave of 16 to 22 Hz with an output of 110 to 115 dB using 2.5 cms H_2_O of pressure. This sound wave has the ability to travel down the tracheobronchial tree and vibrate tracheobronchial secretions. This vibration enhances mucociliary clearance of the lower respiratory tract thereby resulting in the induction of sputum. This functionality of the Lung Flute has been applied to sputum induction for diagnostic testing and for the enhancement of mucus clearance from the lower airways (Data on file, Medical Acoustics) [7],[8]. The Lung Flute is currently FDA approved and available for patient use by a health care provider prescription for both these purposes.

Therapeutic use of the Lung Flute was initially tested in a trial that as designed to meet regulatory requirements. In a FDA 510(k) non-inferiority study, the Lung Flute was compared to a FDA cleared OPEP device (Acapella®) in a eight-week, randomized, controlled, two arm open-label parallel study in 40 COPD patients with chronic bronchitis. Both devices improved COPD symptoms and disease specific health status, with trends favoring the Lung Flute (Data on file, Medical Acoustics). We wanted to confirm and explore further the therapeutic use of the Lung Flute in COPD in a longer trial and compare it to usual care. This report describes the results of such a 26 week study where patients were randomized to the Lung Flute or usual care. The primary endpoint was COPD symptoms as assessed by the Chronic COPD Questionnaire (CCQ) [9]. Secondary endpoints assessed included spirometric lung function, exercise tolerance, exacerbations and health status.

## Methods

### Study design

This was a 26-week, two arm, open label, parallel groups study. Subjects were randomized to the Lung Flute or usual care. The clinical trial registration number of this trial is NCT01186822. The study was approved by the Human Studies subcommittee for the Department of Veterans Affairs (VA) Western New York Healthcare system. All participants provided written informed consent prior to any study procedures.

### Subjects

We had planned to enroll 80 subjects (see power analysis below) with COPD with chronic bronchitis at a single center (Buffalo VA Medical Center). Inclusion criteria were: a) between 30-80 yrs of age, b) presence of airflow obstruction by spirometry post-bronchodilator forced expiratory volume in 1 second (FEV_1_) to forced vital capacity (FVC) ratio <70% and FEV_1_ < 80% predicted, GOLD Stage 2-4), c) presence of chronic bronchitis, i.e. cough productive of sputum on most days of the week, d) current smoker or ex-smoker with at least 10 pack yrs of smoking history, e) able to vibrate the reed of the Lung Flute® using the standard therapeutic maneuver. Exclusion criteria were a) exacerbation of COPD within 4 weeks prior to enrollment, b) predominant asthma and bronchiectasis by clinical assessment, c) pregnant or nursing women d) chronic use of a mucolytic medication.

### Procedures

The study consisted of a screening and randomization visit and then on treatment clinic visits at 2, 14 and 26 weeks. All visits were performed on an outpatient basis. In addition, standardized telephonic assessments were made at 8 and 20 weeks. Participants who met the inclusion/exclusion criteria were randomized to either the Lung Flute or usual care on a 1:1 allocation basis. Randomization was by predetermined random sequence generated independently and kept in sealed envelopes until the time of randomization.

At baseline prior to randomization, symptoms were measured with the CCQ and health status with the St. George’s respiratory questionnaire (SGRQ) [9]-[11]. Lung function was assessed by post-bronchodilator spirometry, exercise capacity by the six minute walk test, dyspnea by the measurement of the modified Medical Research Council (mMRC) score and body mass by calculating the body mass index (BMI). These measurements were used to calculate the BODE index as has been described earlier [12].

At each subsequent clinic visit, all the above evaluations were repeated and review of concomitant medication, compliance assessment, adverse event surveillance and exacerbation history were performed. The telephone calls consisted of a standardized assessment of concomitant medication, compliance assessment, adverse event surveillance and exacerbation history.

### Intervention

The Lung Flute arm participants were instructed to blow twice in to the Lung Flute device vigorously enough to make the reed oscillate, followed by 5 normal breaths. This was repeated 10 times, followed by 3 huff coughs to complete 1 cycle. Two such cycles were recommended twice a day. One of these cycles was performed under supervision of the study personnel at the time of enrollment and at each subsequent study visit. Baseline COPD medication regimen was continued in all participants, although the primary physicians of the participants could make medically necessary changes. Chest physical therapy, additional breathing exercises and formal pulmonary rehabilitation programs were not prescribed to any of the participants during the study.

### Endpoints

The primary endpoint of this study was comparison of the change in COPD symptoms assessed by the CCQ questionnaire between the intervention and control group at 26 weeks. Secondary endpoints included comparison of the changes in SGRQ score, spirometric lung function, BODE index, and exacerbation frequency at 26 weeks between the 2 arms.

### Data analysis

All analyses were performed in an intention to treat (ITT) manner. Subject demographics were compared with t tests and chi square analyses as appropriate. For normally distributed outcomes (e.g. CCQ and SGRQ scores), paired t-test was applied to compare week 26 (week 14/ week 2) to baseline for the two arms, respectively. Also 2-sample independent t-test is used to compare the two arms at baseline, and to compare the changes of week 26 (week 14/ week 2) from baseline in the two arms. For outcomes which failed the normality test (e.g. BODE score), Wilcoxon signed ranks test is applied to compare week 26 (week 14/ week 2) to baseline for the two arms, respectively. Also Wilcoxon-Mann Whitney test is used to compare the two arms at baseline, and to compare the changes of week 26 (week 14/week 2) from baseline in the two arms. Fisher’s exact test is applied to compare the exacerbation frequencies of the two arms. A p < 0.05 was considered significant. Missing data were imputed by carrying forward the last observation.

### Power analysis

In order to determine sample size, we used the primary outcome of the CCQ score. In previous 8 week studies with Lung Flute, an average improvement of 0.4 (S.D. of 0.64) in the CCQ score has been seen with this device. We assumed that the control group will have a change of .05 in the CCQ score. With an alpha = 0.1 (one sided t test) and power = 0.8 and a 1:1 allocation, a total sample size of 76 was required. Allowing for a 5% drop-out rate, a total of 80 patients were to be included in this study.

## Results

### Participants

**Figure 1:**
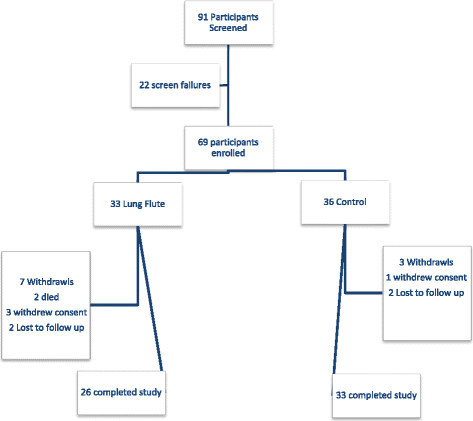
Flow chart of participants in the study.

We screened a total of 91 patients and 69 patients were enrolled (Figure 1). The most common reason for screen failure was the absence of at least moderate airflow obstruction. Of the enrolled patients, 33 were randomized to Lung Flute, while 36 to control arm. Of the enrolled participants 59 completed the study, 26 of the 33 participants in the Lung Flute arm and 33 of the 36 participants in the control arm. None of the early termination/withdrawals were related to the device, except for one patient who withdrew consent at 2 weeks because of perceived lack of efficacy.

Baseline clinical characteristics of the participants randomized in to the study are described in Table 1. There were no statistically significant differences in the demographics, smoke exposure history and lung function between the subjects enrolled in the two arms.

**Table 1:** Subject demographics

**Characteristic**	**Lung Flute (n = 33)**	**Control (n = 36)**	**p value**
Age	68.88 ± 1.49	67.97 ± 1.29	0.58
Gender	Male = 29	Male = 32	0.89
	Female = 4	Female = 4	
Race	Caucasian = 30	Caucasian = 33	0.91
	African-American = 3	African-American = 3	
Smoking history ( pack yrs)	62.16 ± 7.13	60.26 ± 4.60	0.82
Smoking status	Current = 13	Current = 12	0.60
	Ex = 20	Ex = 24	
FEV_1_ (liters)	1.71 ± 0.13	1.53 ± 0.11	0.30
FEV_1_% predicted	51.19 ± 2.98	49.13 ± 3.18	0.64
Baseline COPD medications N (%)			
- LAMA + LABA/ICS	11 (33.3)	19 (52.8)	0.31
- LABA/ICS	6 (18.2)	3 (8.3)	
- LAMA/LABA	2 (6.1)	1 (2.8)	
- LAMA	3 (9.0)	3 (8.3)	
- LABA	1 (3.0)	3 (8.3)	
- SAMA/SABA	5 (15.2)	6 (16.7)	
- SABA/none	5 (15.2)	1 (2.8)	

LAMA = long actiing anti-muscarinic agent (e.g. tiotropium), LABA = long acting β agonist (e.g. formoterol), ICS = inhaled corticosteroid (e.g. budesonide), SAMA = short acting anti-muscarinic agent (e.g. ipratropium), SABA = short acting β agonist (e.g. albuterol).

### COPD symptoms (CCQ)

**Figure 2:**
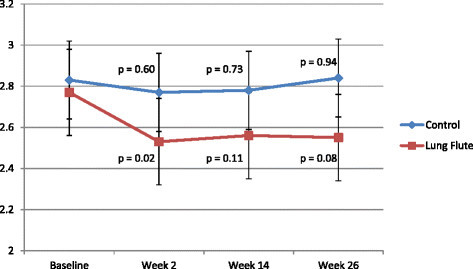
**Change in the CCQ score during the study in the two arms.** The p values are for the change from baseline within each group.

The CCQ is an objective validated tool to assess COPD symptoms [9]. It consists of 10 items, divided into 3 domains: symptoms (4 items), functional state (4 items) and mental state (2 items). Each question can be answered from 0 = best, 6 = worst, and the CCQ score is derived as the average of the individual question scores, with a range of 0-10. A reduction in the CCQ score denotes a reduction in COPD symptoms. There was no difference in the baseline CCQ score in the 2 groups (p = 0.81). The CCQ score at 26 weeks in the control group had increased (worsened) by 0.01 points (p = ns). In the Lung Flute group, a decrease (improvement) of 0.23 points in the CCQ score was seen at 26 weeks (p = 0.08). When intermediate timepoints were examined, the change in CCQ with the Lung Flute was evident at 2 weeks (0.24 points, p = 0.02) and sustained at 14 weeks (0.21 points, p = 0.11) while the changes in the control group were small and non-significant (Figure 2).

When changes in the domain scores were examined in the 2 arms, the symptoms domain showed the largest responses to the lung flute, with a 0.42 point improvement seen at 26 weeks (p = 0.004), as well as significant improvements at 2 and 14 weeks (Tables 2 and 3). The items in this domain quantify shortness of breath at rest, shortness of breath with physical activity, cough and phlegm production respectively. Changes in function and mental domains with the Lung Flute were not consistent or statistically significant.

**Table 2:** Change in CCQ total and domain scores from baseline at 2, 12 and 26 weeks for the Lung Flute group

**Lung Flute group**			
**Score**	**2 weeks**	**14 weeks**	**26 weeks**
Total	-0.24^#^	-0.21	-0.23^##^
Symptom domain	-0.30*	-0.27**	-0.42***
Mental domain	-0.32	-0.17	-0.05
Function domain	-0.14	-0.17	-0.12

^#^p = 0.02, ^##^p = 0.08, *p = 0.003, **p = 0.06, ***p = 0.004.

P values <0.10 are shown.

**Table 3:** Change in CCQ total and domain scores from baseline at 2, 12 and 26 weeks for the control group

**Control group**			
**Score**	**2 weeks**	**14 weeks**	**26 weeks**
Total	-0.06	-0.05	+0.01
Symptom domain	-0.21	-0.14	-0.11
Mental domain	0.0	0.0	0.0
Function domain	+0.05	+0.02	+0.14

P values <0.10 are shown.

### Health status (SGRQ)

**Figure 3:**
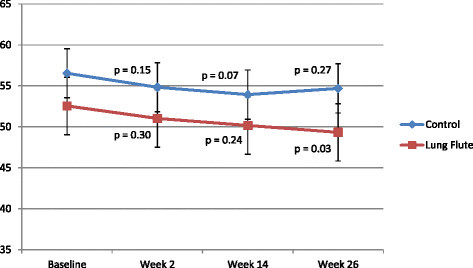
**Change in the SGRQ score during the study in the two arms.** The p values are for the change from baseline within each group.

SGRQ is a well validated, widely used health status questionnaire specific for COPD [11]. A reduction in the SGRQ score denotes an improvement in disease specific health status or quality of life. There was no difference in the baseline SGRQ score in the 2 groups (p = 0.22). The Lung Flute arm showed a progressive improvement in health status, with a significant decrease of 3.23 points seen by week 26 (p = 0.03) (Figure 3). In contrast, the decrease in SGRQ of 1.85 points at week 26 with usual care was non-significant. Consistent with the CCQ observations, the largest and most consistent changes with the Lung Flute were seen in the symptoms domain, with minimal changes in activity and only a 26 week change in the impact domain (Tables 4 and 5). Usual care was not associated with significant improvement in any SGRQ domain.

**Table 4:** Change in SGRQ total and domain scores at 2, 12 and 26 weeks for the Lung Flute group

**Lung Flute group**			
**Score**	**2 weeks**	**14 weeks**	**26 weeks**
Total	-1.52	-2.38	-3.23^#^
Symptom domain	-2.2	-4.1*	-3.6**
Impact domain	-2.25	-1.1	-3.37***
Activity domain	+0.59	-3.34	-1.18

P values <0.10 are shown.

^#^p = 0.03, *p = 0.08, **p = 0.08, ***p = 0.09.

**Table 5:** Change in SGRQ total and domain scores at 2, 12 and 26 weeks for the control group

**Control group**			
**Score**	**2 weeks**	**14 weeks**	**26 weeks**
Total	-1.71	-2.60^#^	-1.85
Symptom domain	-3.6*	-1.3	-2.8
Impact domain	-1.88	-3.1	-1.9
Activity domain	-0.81	-3.36	-1.2

P values <0.10 are shown.

^#^p = 0.07, *p = 0.06.

### BODE score and its components

Over the 26 weeks, BODE score in the Lung Flute arm did not change from baseline, being 3.16 at baseline and 3.16 at 26 weeks. However, in the usual care arm, there was a noticeable worsening in the BODE score, with an increase from the baseline value of 3.31 to 4.14 at 26 weeks (p = 0.0006). When individual components of the BODE score were examined, BMI and FEV_1_ did not change significantly in either arm, however, deteriorations in mMRC and 6 minute walk distances were seen only in the control arm (Tables 6 and 7).

**Table 6:** BODE scores and its components at 2, 12 and 26 weeks for the Lung Flute group

**Lung Flute group**				
**Parameter**	**Baseline**	**2 weeks**	**14 weeks**	**26 weeks**
BODE	3.16 ± 0.49	3.13 ± 0.49	3.25 ± 0.48	3.16 ± 0.45
BMI	27.98 ± 1.19	27.57 ± 1.15	26.27 ± 1.03	26.12 ± 0.97
FEV1% predicted	51.19 ± 2.98	51.19 ± 3.38	50.36 ± 3.24	50.15 ± 3.10
mMRC score	1.48 ± 0.19	1.42 ± 0.20	1.36 ± 0.21	1.52 ± 0.21
6 minute walk distance	353.03 ± 29.95	355.88 ± 32.05	355.63 ± 30.58	359.18 ± 29.19

P values <0.10 are shown.

**Table 7:** BODE scores and its components at 2, 12 and 26 weeks for the control group

**Control group**				
**Parameter**	**Baseline**	**2 weeks**	**14 weeks**	**26 weeks**
BODE	3.31 ± 0.45	3.49 ± 0.50	3.54 ± 0.47	4.14 ± 0.51*
BMI	27.15 ± 0.74	27.10 ± 0.74	27.14 ± 0.73	26.91 ± 0.73
FEV1% predicted	49.13 ± 3.18	47.60 ± 3.31	46.82 ± 3.30***	47.16 ± 3.45
mMRC score	1.81 ± 0.15	1.81 ± 0.18	1.83 ± 0.19	2.22 ± 0.19**
6 minute walk distance	364.20 ± 25.73	353.47 ± 28.35	358.21 ± 26.52	322.41 ± 29.78 ****

P values <0.10 are shown.

*p = 0.0006, **p = 0.01, ***p = 0.03, ****p = 0.007.

### Exacerbations

**Figure 4:**
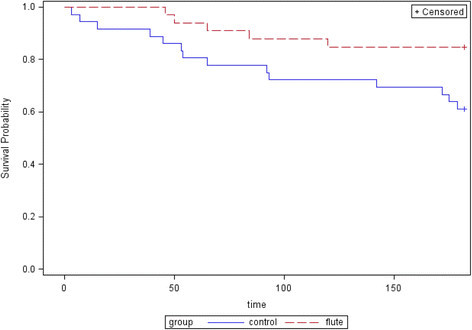
Probability of not having an exacerbation during the study in the two arms (p = 0.03, Wilcoxon signed rank test).

Six of the 33 patients in the Lung Flute group, while 14 of 36 patients in the control group experienced at least one moderate to severe exacerbation, defined as an exacerbation requiring outpatient treatment with antibiotics and/or corticosteroids or requiring hospitalization (p = 0.07). Figure 4 illustrates the timing of the first exacerbation during the study in the 2 arms, and the probability of not having an exacerbation favors the lung flute (p = 0.03).

### Patient preference

When asked at the end of the study visit, 85% of the patients that were randomized to the lung flute indicated that they found the device to be efficacious and would like to continue using it as a regular part of their COPD care regimen.

### Safety

There were two deaths in the Lung Flute group and none in the control group. There were serious adverse events in both groups, most commonly hospitalization due to COPD or co-morbid conditions. None of the deaths and serious adverse events were determined to be related to Lung Flute use. One patient complained of increased cough with the Lung Flute. There were no study withdrawals related to the adverse effects with the use of the Lung Flute.

## Discussion

There is an unmet medical need for dealing with mucus hypersecretion, impaired mucociliary clearance and secretion retention in COPD, with a paucity of treatments that have demonstrated efficacy for these disorders. Currently available mucolytics and expectorants are of unproven efficacy in COPD, and the beneficial effects of agents such as n-acetylcysteine and carbocisteine are more likely related to their antioxidant effects rather than their mucolytic effects [13],[14]. Mechanical means to improve mucus clearance in hypersecretory lung conditions include Oscillatory PEP devices such as the Acapella and Flutter, chest vibration and percussion and breathing techniques. However, these have not been tested systematically in stable COPD. This post-marketing study confirms the previous regulatory study that the Lung Flute is efficacious in COPD with chronic bronchitis in improving respiratory symptoms and health status. The largest improvements were seen in symptom domains of the CCQ and SGRQ. Furthermore, it confirms the safety of this device in COPD with related adverse effects being seldom seen.

In addition to the improvements in symptoms and health status, several of the secondary endpoints also demonstrated the benefit of the Lung Flute in COPD. The Lung Flute appeared to stabilize the BODE score, primarily through preventing the progressive decrement in 6 minute walk distance and increase in dyspnea seen in COPD. Furthermore, a trend to reduction of exacerbations was seen. These observations need to be confirmed in the future with studies that are of appropriate size and duration to definitively assess treatment benefit in these aspects of COPD.

The mechanism of action that results in clinical benefits of Lung Flute in COPD is presumed to be increased mucocilary clearance of tracheobronchial secretions. However, a limitation of this study was the absence of direct measurement of mucociliary clearance. Other limitations of this study include it being a single site study of relatively small size, and the lack of blinding, which is difficult in device studies. However, of note, all endpoints in this study were either patient reported outcomes (CCQ, SGRQ, mMRC) or were objective measures (Spirometry, 6 minute walk distance). Subjective investigator assessments of treatment effects were therefore avoided in this trial, minimizing bias. Furthermore, the control group clearly showed either no change or slight worsening in all these parameters as expected.

We had planned to enroll 80 participants in the study but a larger than expected screen failure rate and financial and time constraints resulted in enrollment of smaller number of 69 participants. The mean magnitude of improvement at 26 weeks in the CCQ with the Lung Flute was also somewhat smaller in the current trial (-0.23) than in the original registration 8 week trial (-0.40). These factors likely explain why the CCQ improvement in the Lung Flute group did not reach statistical significance. However, the totality of the benefit seen in the various other endpoints assessed does support the efficacy of the Lung Flute in COPD with Chronic Bronchitis.

## Conclusions

The cost of chronic care of COPD continues to grow with expansion of the possible medication regimen that could be used in these patients. Each additional medication also places the patient at increased risk of adverse effects. Furthermore, none of the standard treatments, inhaled bronchodilators, inhaled corticosteroids or phosphodiestrase inhibitors have demonstrated effects to improve mucociliary clearance in COPD. The Lung Flute therefore represents a welcome addition to our armamentarium for the treatment of COPD because of its potential unique mechanism of action, low cost and safety.

## Abbreviations

COPD: Chronic obstructive pulmonary disease

CCQ: Chronic COPD Questionnaire

SGRQ: St. George Respiratory questionnaire

FDA: Food and Drug Administration

OPEP: Oscillatory Positive Expiratory Pressure

VA: Veterans Affairs

FEV_1_: Forced expiratory volume in 1 second

FVC: Forced vital capacity

mMRC: Modified Medical Research Council

BMI: Body mass index

ITT: Intention to treat

## Competing interests

The authors declare that they have no competing interests.

## Authors’ contributions

SS: was the principal investigator for this study. He participated in study design, oversaw the execution and performed the data analysis and manuscript preparation. JY; was the biostatistician and performed the statistical analysis and assisted in manuscript preparation. PA: was the study coordinator for the trial and enrolled and followed up patients and collected all relevant data. All authors read and approved the final manuscript.
